# Anti-Nuclear Antibody (ANA) Positivity and Nuclear Antigen Reactivity in Patients with Joint Hypermobility Syndrome/Hypermobile Ehlers Danlos Syndrome (JHS/hEDS)

**DOI:** 10.3390/biomedicines13092134

**Published:** 2025-09-01

**Authors:** Lindsay Moy, Aleksander Lenert, Petar Lenert

**Affiliations:** Division of Immunology, Department of Internal Medicine, The University of Iowa, 200 Hawkins Drive, Iowa City, IA 52242, USA; lindsaynmoy@gmail.com (L.M.); aleksander-lenert@uiowa.edu (A.L.)

**Keywords:** anti-nuclear antibody, joint hypermobility syndrome, hypermobile Ehlers Danlos Syndrome

## Abstract

**Background/Objectives**: To compare clinical features of patients with joint hypermobility syndrome/hypermobile Ehlers Danlos Syndrome (JHS/hEDS) who tested positive or negative for anti-nuclear antibodies (ANA), and to determine antibody titers, staining patterns, and reactivity to common nuclear autoantigens. **Methods**: ANA results were determined by Hep2 immunofluorescence assay. Reactivity to the most common nuclear autoantigens was measured by the Multiplex assay. Clinical manifestations were compared between three subgroups: total ANA+, ANA+ who did not have evidence of systemic autoimmune inflammatory disease (SAID), and ANA−. **Results**: Of 289 patients, 210 patients had a Beighton score > 5 and were tested for ANA antibodies. One hundred and thirty-one patients had a positive ANA test. Twenty patients in this subgroup were classified as SAID+ while the remaining 111 patients did not meet criteria for any systemic disease. Speckled staining was the most observed pattern in both ANA+SAID+ (75.00%) and ANA+SAID− (72.97%) subgroups. In the latter subgroup, the target of nuclear autoreactivity remained elusive in 80% of patients. The most common clinical manifestations were diffuse arthralgias, myofascial pain, sicca symptoms, Raynaud’s phenomenon, gastrointestinal manifestations, and chronic fatigue. Joint dislocations were observed more commonly in the ANA− subgroup compared to ANA+SAID− patients (30.38% vs. 12.61%, adjusted *p* < 0.05). **Conclusions**: Similar clinical characteristics were observed in ANA+ and ANA− subgroups of JHS/hEDS, except for joint dislocations which were more common in the ANA− subgroup. The target of ANA reactivity was unknown in 80% of ANA+JHS/hEDS patients and needs to be determined in future studies.

## 1. Introduction

Joint hypermobility is a clinical term used to describe individuals who are colloquially labelled as “double-jointed.” Historically, individuals who possessed this trait and experienced related sequelae were considered to have joint hypermobility syndrome (JHS). The early criteria for JHS were largely based on the Beighton scoring system [1]. Individuals were scored based on physical exam findings and could receive a maximum score of nine points.

Joint hypermobility has also been recognized as the predominant feature of hypermobile Ehlers Danlos Syndrome (hEDS). hEDS is one of the most common heritable connective tissue disorders, characterized by joint hypermobility and skin hyperextensibility [2]. Unfortunately, while other subtypes of EDS benefit from genetic testing, the underlying genetic basis of hEDS remains unidentified [2]. The diagnostic criteria for hEDS have been modified over time [3]. The most recent criteria published in 2017 require each of the following: (1) the presence of generalized joint hypermobility (identified by Beighton scoring); (2) two or more of the following: (a) systemic manifestations of a genetic connective tissue disorder, (b) positive family history in a first degree relative, (c) musculoskeletal complications; and (3) absence of unusual skin fragility, exclusion of other heritable and acquired connective tissue disorders, and exclusion of alternative diagnoses that may include joint hypermobility [3]. As alluded to in the 2017 hEDS diagnostic criteria, these individuals typically experience systemic manifestations and can present with a variety of symptoms [3]. Given significant overlap in symptomatology, JHS and hEDS are no longer recognized as separate entities as they are clinically indistinguishable [3,4].

The prevalence of patients with symptomatic hypermobility is high in the general population and remains under-recognized [4,5]. As access to genetic counselors is variable, non-standardized, and frequently delayed, many of these patients are referred to rheumatology specialists instead, with some institutions even requiring a rheumatologic evaluation prior to genetics referral [4].

When our cohort of hypermobile patients was analyzed, we noticed that a high proportion of referred patients had a positive anti-nuclear antibody (ANA) test. It prompted us to question whether hypermobile patients with a positive ANA test may have a different clinical phenotype and whether they should be managed differently. With a potential underlying genetic component contributing to their enhanced joint flexibility, we wondered whether these hypermobile patients would be more likely to develop an autoimmune connective tissue disease [6]. Should they be followed more closely or for longer periods of time compared to positive ANA patients without joint hypermobility?

The main objective of this study was to compare clinical manifestations in JHS/hEDS patients who tested ANA positive to those who tested ANA negative. We further sought to analyze autoantibody profiles in patients who tested ANA positive and evaluate for reactivity to the most common nuclear autoantigens.

## 2. Materials and Methods

### 2.1. Study Design and Population

This was a retrospective cross-sectional unmatched single center comparison study. This study included a total number of 289 consecutive patients with JHS/hEDS who were evaluated at the University of Iowa Adult Rheumatology Clinic. Patients were referred to rheumatology between 2000 and 2024 for evaluation of suspected connective tissue disease given various musculoskeletal complaints.

A detailed evaluation included determination of demographic characteristics and measurement of the Beighton score. The presence or absence of the following clinical manifestations were assessed: arthralgias (affecting small joints, large joints, and specifically the patellofemoral compartments), myofascial pains, fatigue, skin laxity, history of joint dislocations, history of easy bruising, dysautonomia (including postural orthostatic tachycardia syndrome [POTS]), gastrointestinal symptoms (including gastroesophageal reflux disease and irritable bowel syndrome), urticaria (including any degree of urticarial-type rash), livedo reticularis, sicca symptoms, ocular problems other than keratoconjunctivitis sicca (including inflammatory eye disorders), Raynaud’s phenomenon (complete and incomplete), and thyroid disease.

Laboratory data included determination of the ANA staining pattern and titer by immunofluorescent microscopy on Hep2 cells (BioPlex 2200 System, BioRad Laboratories, Hercules, CA, USA). In those who tested ANA positive, reactivity to most common antinuclear antibody targets was analyzed. We measured the serum concentration of anti-dsDNA, anti-Smith, anti-U1 RNP, anti-Smith/RNP common motif, anti-SS-A (60 kD), anti-SS-B, and anti-histone antibodies. In patients who tested positive for anti-dsDNA antibodies, further confirmatory immunofluorescent testing on slides with immobilized *Crithidia luciliae* cells was performed. Kinetoplast staining was considered a positive test. In a smaller subset of patients with Raynaud’s phenomenon, further testing against anti-centromere, anti-Scl-70 (topoisomerase I), and anti-RNA polymerase III was performed. Solid phase Multiplex assay (BioPlex 2200 System, BioRad Laboratories, Hercules, CA, USA) was used for measurement.

**Figure 1:**
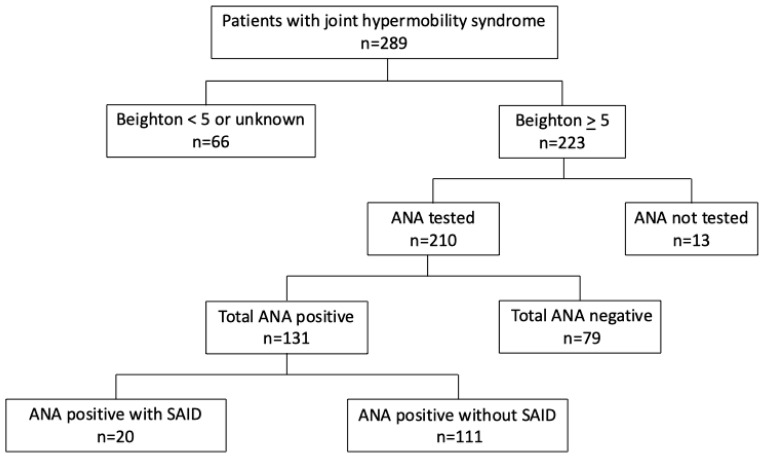
Study population. Study population divided by Beighton score, ANA result, and presence of definitive systemic autoimmune disease. ANA = anti-nuclear antibody, SAID = systemic autoimmune disease.

Based on the commonly recognized definition of JHS/hEDS as a Beighton score of five or above, we narrowed down our initial cohort from 289 to 223 patients (Figure 1). We further excluded those patients who did not have an ANA test performed, which brought our cohort down to 210 patients. Of the 210 patients in which ANA results were available, a positive ANA test (ANA+) was found in 131 patients and a negative ANA test (ANA−) in 79 patients. Within the ANA+ group, 20 patients met criteria for definitive systemic autoimmune disease (SAID) and were analyzed separately from the remaining cohort of 111 ANA+SAID− patients (Figure 1). The SAID diagnoses included SLE, MCTD, SjS, pauci-articular JIA, sarcoidosis, polymyositis, primary biliary cirrhosis, and HLA-B27+ oligoarticular asymmetric inflammatory spondylarthritis.

The study was approved by the Institutional Review Board at the University of Iowa Hospitals and Clinics (approval number IRB#: 202003670). Patient consent was waived as this was a retrospective chart review analysis without intervention.

### 2.2. Data Analysis

In this cross-sectional study, we calculated summary statistics of our study sample. For continuous variables, we calculated means with standard deviation (SD). For categorical variables, we presented counts with percentages (%). Between-group analyses of categorical variables (clinical manifestations and reactivity to nuclear antigens) were performed with the Chi-square or Fisher’s exact tests. We used the Benjamini–Hochberg procedure to correct for multiple testing and we considered an adjusted *p*-value < 0.05 as significant [7]. All analyses were performed with SAS (version 9.4).

## 3. Results

### 3.1. Demographic Characteristics

At initial rheumatologic evaluation, the overwhelming majority of our cohort were young females in their 30s. When total ANA+, ANA+SAID−, and ANA− subgroups were compared, their baseline demographics were quite similar (Table 1).

### 3.2. Hypermobility Assessment

The mean Beighton score was similar across all study groups (Table 1).

### 3.3. Clinical Manifestations

When comparing arthralgias across groups, there was no statistically significant difference. We further subdivided arthralgias into large and small joint groups, as certain rheumatic diseases have a predilection for specific joint sizes; however, this did not result in any notable difference. Patellofemoral pain, myofascial pain, fatigue, skin laxity, easy bruising, POTS, gastrointestinal symptoms, urticaria, livedo reticularis, sicca symptoms, ocular disease (excluding keratoconjunctivitis sicca), Raynaud’s phenomenon, and thyroid disease were also not significantly different across groups. Interestingly, we observed a higher proportion of joint dislocations in the ANA− subgroup compared to the ANA+SAID− subgroup (Table 1, adjusted *p* < 0.05).

### 3.4. Ana Results

**Figure 2:**
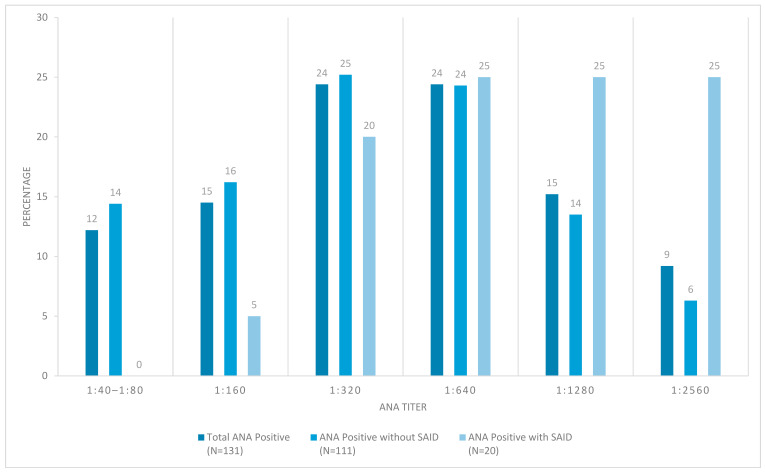
ANA titers in patients with joint hypermobility syndrome. Different subgroups of positive ANA patients by titer. ANA = anti-nuclear antibody, SAID = systemic autoimmune disease.

**Figure 3:**
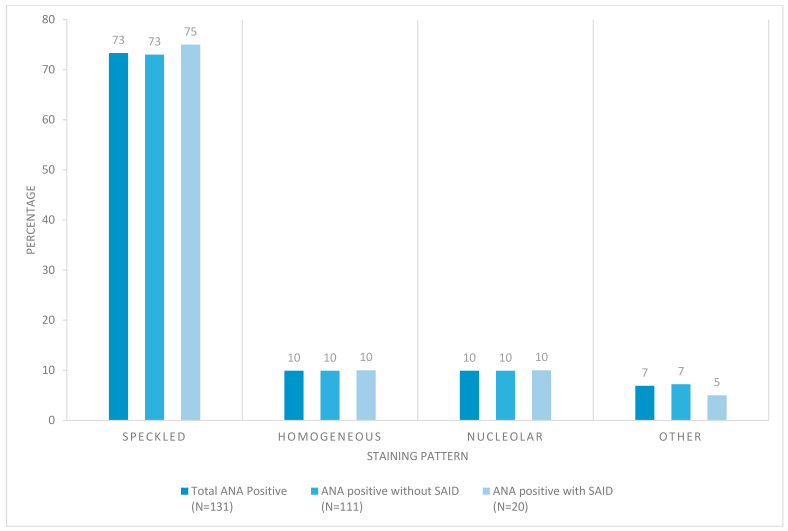
ANA staining pattern in patients with joint hypermobility syndrome. Different subgroups of positive ANA patients by ANA pattern. ANA = anti-nuclear antibody, SAID = systemic autoimmune disease.

Out of the 131 ANA+ patients, the majority of patients had moderate to high ANA titers. As one may expect, those with definitive SAID tended to have higher ANA titers (Figure 2). Comparing the staining patterns, the majority of ANA+ patients (both SAID positive and SAID negative subgroups) showed a speckled pattern. Both coarse and fine speckled patterns were observed, followed by similar percentages of homogeneous and nucleolar patterns (Figure 3).

### 3.5. Autoantibody Reactivity to Specific Nuclear Autoantigens

Autoantibody targets commonly associated with autoimmune connective tissue diseases (SLE, MCTD, SjS, systemic sclerosis, and drug-induced lupus) such as anti-dsDNA, anti-Smith, anti-U1 RNP, anti-Smith/RNP common motif, anti-SS-A (60 kD), anti-SS-B, anti-centromere, anti-Scl-70 (topoisomerase I), anti-RNA polymerase III, and anti-histone antibodies were then compared between ANA+SAID+ and ANA+SAID− subgroups (Table 2). ANA+SAID+ patients were more likely to react to dsDNA, Smith, U1-RNP, Smith/RNP, SS-A and SS-B antibodies. Autoantigen reactivity remained unknown in 79.81% of patients in the ANA+SAID− subgroup versus 35.00% of patients in the ANA+SAID+ subgroup (adjusted *p* < 0.05 by Fisher’s exact test).

**Table 2:** Reactivity against select nuclear autoantigens in patients with joint hypermobility syndrome.

	Total ANA Positive (N = 131)	ANA Positive Without SAID (N = 111)	ANA Positive with SAID * (N = 20)
	number of positive results/total number tested (%)		
dsDNA	5/101 (5)	1/86 (1)	4/15 (27)
Smith	4/101 (4)	1/85 (1)	3/16 (19)
U1-RNP	8/90 (9)	4/79 (5)	4/11 (36)
Smith/RNP	2/52 (4)	0/45 (0)	2/7 (29)
SS-A	10/111 (9)	5/95 (5)	5/16 (31)
SS-B	5/111 (5)	2/95 (2)	3/16 (19)
Centromere	1/10 (10)	1/10 (10)	n.t.
Scl-70	1/57 (2)	1/52 (2)	0/5 (0)
RNA pol III	1/11 (9)	1/11 (9)	n.t.
Histone	5/17 (29)	5/15 (33)	0/2 (0)
Unknown	90/124 (73)	83/104 (80)	7/20 (35)

n.t. = not tested. SAID = systemic autoimmune disease. * ANA positive with SAID compared to ANA positive without SAID, adjusted *p* < 0.05 (Fisher’s exact test).

### 3.6. Additional Analyses

We further subdivided our total ANA+ patients into different subgroups based on ANA titer and found similar results for age at initial rheumatologic presentation and Beighton scoring (Table 3). Again, no difference in clinical manifestations was observed between these subgroups.

## 4. Discussion

The major objective of our study was to analyze clinical characteristics in patients with JHS/hEDS who were referred to rheumatology and who tested either ANA+ or ANA−. Interestingly, our cohort population was comprised of predominantly young females in their mid-30s with similar underlying joint hypermobility, as evidenced by a Beighton score of approximately 7 and comparable clinical symptomatology. Patients had a constellation of symptoms that were suggestive of a pain amplification syndrome with polyarthralgia, myofascial pain, and fatigue. Other features that were common amongst patients with JHS/hEDS included patellofemoral pain, dysautonomia (i.e., POTS), gastrointestinal symptoms (i.e., gastroesophageal reflux disease, irritable bowel syndrome), and cutaneous elements (i.e., skin laxity, easy bruising, urticaria, livedo reticularis).

Previous studies have identified JHS/hEDS as a cause of chronic pain, and given extensive clinical similarities, JHS/hEDS may even be classifiable as part of the fibromyalgia spectrum [5,6,8]. For example, in a study conducted on 59 children with primary fibromyalgia syndrome, joint hypermobility was found in 14% of patients while 28.8% of patients tested ANA positive [8]. Prior investigations have also described an association between dysautonomia and JHS/hEDS as well as gastrointestinal disorders and JHS/hEDS [9,10,11]. Furthermore, urticaria and mast cell activation syndrome have been linked to JHS/hEDS [11,12,13]. Given the high frequency of these symptoms identified in JHS/hEDS patients, there appears to be a discernible link, yet the principal questions remain unanswered: what is the true mechanistic relationship between JHS/hEDS and these ailments and what is the underlying pathophysiology?

In the same vein, studies have described a strong association between ANA positivity and fatigue, even in those lacking a SAID diagnosis [14]. Dinerman et al. studied the incidence of ANA, Raynaud’s, and sicca symptoms in a cohort of 118 patients with fibromyalgia syndrome and suggested a link between fibromyalgia and connective tissue diseases [15]. More recently, patients with post-COVID-19 syndrome were found to have a high prevalence of fibromyalgia. These patients were usually of female gender, had evidence of joint hypermobility, and tended to test positive for ANA antibodies [6]. The same group suggested that joint hypermobility syndrome and fibromyalgia may overlap in up to 80% of patients.

With ANA testing having a high sensitivity (98%) for SLE, rheumatologists are most often the clinicians tasked with determining the significance of a positive ANA result [16]. It has been shown that there is no increased risk of progression to SAID in ANA+ patients with fatigue and fibromyalgia-like symptoms [15,16]. Interestingly, the more recent literature suggests there may be a component of immune dysregulation in the pathogenesis of fibromyalgia [17].

With this study, we introduce the additional variable of joint hypermobility with its presumed, yet to be defined, genetic basis. A considerable percentage of JHS/hEDS patients tested ANA positive even at very high ANA titers without an active identifiable systemic autoimmune disease process. These results pose the question of whether a systemic disease may arise in the future.

Though we did not find an apparent association between ANA positivity and JHS/hEDS, we recognize that as a retrospective analysis, our study has several limitations and there are likely confounding factors that restrict our ability to draw definitive conclusions. However, to our knowledge, this is the first study to evaluate a potential association between JHS/hEDS and ANA positivity.

## 5. Conclusions

Patients with JHS/hEDS present with a variety of clinical manifestations, including chronic joint and muscle pains, fatigue, Raynaud’s, sicca syndrome, dysautonomia, skin manifestations, and gastrointestinal problems. Surprisingly, within our cohort of JHS/hEDS patients, a substantial percentage of them had a positive ANA test result. Subsequent analysis failed to identify the autoantigen target in about 80% of patients. When ANA+ and ANA− subsets were compared, the only clinical manifestation that reached clinical significance was higher incidence of joint dislocations in the ANA− subgroup. Future studies should be carefully planned to prospectively follow these patients and estimate their risk of progression to a well-defined systemic autoimmune disease.

## Figures and Tables

**Table 1:** Demographics and clinical manifestations in patients with joint hypermobility syndrome.

	Total ANA Positive	ANA Positive Without SAID	Total ANA Negative
	(N = 131)	(N = 111)	(N = 79)
Demographic characteristics			
Female sex at birth	130 (99)	110 (99)	73 (92)
Age at initial evaluation, mean (SD) years	34 (10)	35 (10)	38 (10)
Hypermobility assessment			
Beighton score, mean (SD)	7 (1)	7 (1)	7 (1)
Clinical manifestations			
Arthralgia, large joints	78 (60)	66 (60)	54 (68)
Arthralgia, small joints	70 (54)	58 (52)	43 (54)
Arthralgia, large & small joints	54 (42)	46 (41)	36 (46)
Patellofemoral pain	28 (22)	25 (23)	16 (20)
Myofascial pain	55 (42)	48 (43)	29 (37)
Fatigue	66 (51)	56 (51)	37 (47)
Skin laxity	15 (12)	14 (13)	12 (15)
Joint dislocations *	16 (12)	14 (13)	24 (30)
Easy bruising	21 (16)	18 (16)	18 (23)
POTS	19 (15)	16 (14)	18 (23)
Gastrointestinal symptoms	42 (32)	39 (35)	24 (30)
Urticaria	12 (9)	11 (10)	7 (9)
Livedo reticularis	19 (15)	15 (14)	17 (22)
Sicca symptoms	45 (35)	38 (34)	29 (37)
Ocular problems †	9 (7)	8 (7)	4 (5)
Raynaud’s phenomenon	43 (33)	35 (32)	17 (22)
Thyroid disease	21 (16)	19 (17)	12 (15)
SAID	20 (15)	0 (0)	0 (0)

SAID = systemic autoimmune disease. * Statistically significant difference between ANA positive without SAID and ANA negative patients, adjusted *p* < 0.05 (Chi-square test). † Excluding keratoconjunctivitis sicca. Categorical variables are presented in numbers with percentages in parentheses.

**Table 3:** Beighton score in different subgroups of patients with joint hypermobility.

	Number of Patients	Age at Initial Evaluation Mean (SD) Years	Beighton Score Mean (SD)
Beighton score ≥ 5	223	36 (11)	7 (1)
ANA tested	210	36 (10)	7 (1)
Total ANA positive	131	34 (10)	7 (1)
ANA positive without SAID	111	35 (10)	7 (1)
ANA positive with SAID	20	32 (11)	7 (1)
ANA positive, titer ≥ 1:320	96	33 (10)	7 (1)
ANA positive without SAID, titer ≥ 1:320	77	34 (9)	7 (1)
ANA positive without SAID, titer ≤ 1:160	34	38 (11)	7 (1)
Total ANA negative	79	38 (10)	7 (1)

SD = standard deviation. SAID = systemic autoimmune disease.

## Data Availability

The original contributions presented in this study are included in the article. Further inquiries can be directed to the corresponding author.

## Abbreviations

The following abbreviations are used in this manuscript:

SLE	Systemic Lupus Erythematosus
MCTD	Mixed Connective Tissue Disease
SjS	Sjogren’s Syndrome
JHS	Joint Hypermobility Syndrome
hEDS	Hypermobile Ehlers Danlos Syndrome
POTS	Postural Orthostatic Tachycardia Syndrome
ANA	Anti-Nuclear Antibody
SD	Standard Deviation
